# Bioinformatics-Driven Identification of p62 as A Crucial Oncogene in Liver Cancer

**DOI:** 10.3389/fonc.2022.923009

**Published:** 2022-06-24

**Authors:** Ling Wang, Culton R. Hensley, Mary E. Howell, Shunbin Ning

**Affiliations:** ^1^ Department of Internal Medicine, Quillen College of Medicine, East Tennessee State University, Johnson City, TN, United States; ^2^ Center of Excellence for Inflammation, Infectious Diseases and Immunity, Quillen College of Medicine, East Tennessee State University, Johnson City, TN, United States

**Keywords:** p62, LIHC, SRX1, TXNRD1, algorithm analysis

## Abstract

Liver hepatocellular carcinoma (LIHC) is the major form of liver cancer that is the fourth most common cause of cancer death worldwide. It has been reported that the multifunctional protein p62 (also known as SQSTM1) plays a cancer-promoting role in LIHC, but the detailed mechanisms underlying p62 interaction with LIHC remains unclear. To gain a comprehensive understanding of p62 interaction with LIHC in clinical settings, we performed bioinformatic analyses using various online algorithms derived from high throughput profiling. Our results indicate that p62 expression is significantly upregulated, partially due to its promoter demethylation, rather than p62 gene mutation, in LIHC. Mutation of TP53, CTNNB1, or ALB significantly correlates with, and mutation of AXIN1 reversely correlates with, the p62 expression level. Its upregulation occurs as early as liver cirrhosis, and go through all stages of the carcinogenesis. HCV infection makes a significant contribution to p62 upregulation in LIHC. We further identified p62-associated molecular signatures in LIHC, including many genes that are involved in antioxidant stress and metabolism, such as SRX1 and TXNRD1. Regarding to the clinical outcome, p62 expression level reversely correlates with the survival of LIHC patients (p<0.01). Importantly, we experimentally validated that p62 depletion in liver cancer cell lines downregulates the expression of SRX1 and TXNRD1 at both transcriptional and translational levels, and reduces cell proliferation. As the potential mechanisms underlying the tumor-promoting role of p62, we show that p62 upregulation is remarkably associated with reprogramming of pathways mediated by p53, Wnt/β-catenin, and Keap1-NRF2, which are crucial for oncogenesis in many contexts. Our findings provide a comprehensive insight into the interaction between p62 and LIHC, offering valuable information for understanding of LIHC pathogenesis.

## Introduction

Liver hepatocellular carcinoma (LIHC), or hepatocellular carcinoma (HCC), is the major form of liver cancer that is the fourth most common cause of cancer death worldwide, with a 5-year survival rate of about 18% (1). LIHC is causally associated with chronic tissue damage and inflammation, and stress and environmental carcinogen exposure (such as obesity and alcohol consumption), with chronic viral infections (HCV and HBV) playing the major role.

Nearly three thousand genetic mutations have been identified in LIHC patients, and the most frequently mutated loci include the TERT promoter and the genes coding for P53, β-Catenin, ALB, KEAP1, and NRF2 (2). Interestingly, both Keap1 and NRF2 are the components of the Keap1-NRF2 pathway that transactivate a pool of approximate 250 target genes, of which many are involved in antioxidant defense including p62 (as known as SQSTM1), Cox-2, iNOS, PRDX1, HIF1, NQO1, HMOX1, GSTs, and Keap1 and NRF2 themselves (3–6).

As a key transcriptional target of the transcription factor NRF2, p62 plays crucial roles in DNA damage response (DDR), cancer development, mTORC1-mediated nutrient sensing and metabolism, cell death, aging, inflammation and immunity, cell differentiation, osteoclastogenesis, neurotrophin properties and obesity, dependently or independenely of the autophagy machinery (2, 7–12).

The tumor-promoting properties of p62 are underscored by the facts that p62 is upregulated in different cancer contexts, including LIHC, and breast and prostate cancers (2, 13–17), and that p62 is induced by the oncoprotein Ras that accounts for more than 25% of human cancers (18). p62 overexpression in LIHC predicts poor prognosis (15). In mouse models with defective autophagy, p62 ablation decreases tumorigenesis (18).

To achieve a comprehensive understanding of the association of p62 with the development of LIHC, in this study, we have employed various online algorithms to conduct secondary analyses of available datasets. We show that p62 expression is significantly upregulated in LIHC, and identified p62-associated molecular signatures in this setting. We experimentally confirmed that p62 depletion in liver cancer cell lines downregulates the expression of SRX1 and TXNRD1 at both transcriptional and translational levels, and reduces cell proliferation. Moreover, these meta-analyses of clinical samples consolidate the claim that p62 can serve as a prognostic marker for LIHC patients.

## Methods

### Algorithm Meta-Analysis

We employed different online algorithms for metadata analysis, including Oncomine (19), Genotype-Tissue Expression (GTEx), Gene Expression Atlas, ProteinAtlas, proteomicsDB, Tumor Immune Estimation Resource (TIMER v2) (20, 21), Gene Expression Profiling Interactive Analysis (GEPIA v2) (22), Tumor-Immune System Interactions Database (TISIDB) (23), UALCAN (24), COSMIC, Tumor Fusion Gene Data Portal (TumorFusions) (25), FusionGDB (26), ChimerDB v4 that integrates several different fusion portals such as STARFusion, TCGA-FAWG, and FusionScan (27), cBioportal (28, 29), DriverDBv3 (30), Kaplan Meier Plotter (KMPlot) (31), muTarget (32), and EMSEMBL, for mRNA and protein expression, correlation, gene mutation, fusion, tumor-immune interaction, and survival analyses. All portals include the Cancer Genome Atlas (TCGA) datasets, in addition to other unique datasets obtained from patients and cell lines. BioGRID (33, 34), GeneMANIA (35, 36), STRING, Uniprot, and KEGG portals were applied for post-translational modifications, signaling pathway, and protein-protein and functional interaction analyses.

All analyses were conducted using the default settings if not otherwise indicated, with the detailed dataset information and guidelines provided online by each portal. p<0.05 is considered statistically significant and >0.05 is non-significant (n.s.), and p<0.01 is considered statistically very significant.

### Cell Lines

Two liver cancer epithelial cell lines, Huh7D12 and HepG2 were used to validate the target genes regulated by p62 at the transcriptional and translational levels. These cell lines were cultured at 37°C with DMEM media plus 5% FBS (the lower percentage was reported to reduce cell aggregation without affecting cell growth) and antibiotics (Life Technologies).

### Lentiviral Transfection and CRISPR-Mediated Depletion

p62-specific CRISPR/Cas9 plasmids were generated by GenScript by cloning p62 sgRNAs into pLenti-CRISPRv2 eSpCas9 lentiviral vector (puro), and the targeting sequences are (both in cDNA): p62 sgRNA#1: 5’-GAAGATGTCATCCTTCACGT, and p62 sgRNA#2: 5’-TTCGGATTCTGGCATCTGTA. Lentivirus packing and transfection, and selection of stable polyclonal transfectants with puromycin were carried out as detailed in our previous publication (37).

### Reagents, Antibodies and Immunoblotting

p62 (clone D-3) mouse monoclonal antibody was from Santa Cruz. Mouse TXNRD1 (clone 1B10C4) and rabbit SRX1 (polyclonal) antibodies were purchased from Proteintech. HRP-coupled secondary antibodies were from Cell Signaling Technologies.

Cell lysates were lysed with NP40 lysis buffer (150 mM NaCl, 1% NP-40, 50 mM Tris-pH 8.0, plus protease inhibitors), followed by immunoblotting (IB) with indicated antibodies, and signals were detected with the enhanced chemiluminescence (ECL) kit following the manufacturer’s protocol (Amersham Pharmacia Biotech).

The broad-spectrum inhibitor of histone demethylases IOX1 was purchased from MedChemExpress.

### Primers and Real-Time qPCR

Total RNA was isolated from the tested liver cancer cell lines with an RNeasy Mini kit (Qiagen). Reverse transcription was performed with an AMV-mediated RT kit (Promega). Quantitative real-time PCR (qPCR) was performed with the use of SYBR Green (Applied Biosystems), on a CFX96™ Real-time PCR Detection System (Bio-Rad). All reactions were run in triplicates. Mean cycle threshold (*C*_t_) values were normalized to 18s rRNA, yielding a normalized C*_t_* (Δ*C*_t_). ΔΔ*C*_t_ value was calculated by subtracting respective control from the Δ*C*_t_, and the expression level was then calculated by 2 raised to the power of respective -ΔΔ*C*_t_ value. The averages of 2^(-ΔΔ*C*_t_) in the control samples were set to 1 or 100%. Results are the average ± standard error (SE) of duplicates or triplicates for each sample. Primers for real-time qPCR are as follows: Txnrd1: F: 5’-GTTACTTGGGCATCCCTGGTGA-3’; R: 5’-CGCACTCCAAAGCGACATAGGA-3’. Srx1: 5’- GCAGAGCCTCGTGGACACGAT-3’; R: 5’-ATGGTCTCTCGCTGCAGTTGCT-3’; HTATIP2: F: 5’- GCCTGTTTTCCAAAGTCACGCTC-3’; R: 5’- CCTTGAAAGGCAGAGGCGTAGT-3’. TTC1: F: 5’- AACATGTCGGATGAAGAGAAACAG-3’; R: 5’- GGAAGCAGGATGGGCACATTTC-3’. p62: F: 5’-CAGGCGCACTACCGCGATG-3’, and R: 5’-ACACAAGTCGTAGTCTGGGCAGAC-3’. 18s rRNA: F: 5’-GGCCCTGTAATTGGAATGAGTC-3’, and R: 5’-CCAAGATCCAACTACGAGCTT-3’.

### Proliferation Assay

MTT proliferation assay was carried out following the manufacturer’s instructions (Promega). Cells were seeded 1X10^5^ per well in 6-well plates. The proliferation rates of the control sgRNA were set to 100%. Data are expressed as mean ± standard error (SE) of triplicate samples, and representative results from at least three independent repeats with similar results are shown.

## Results

### Tissue- and Cell-Specific Expression of p62

**Figure 1:**
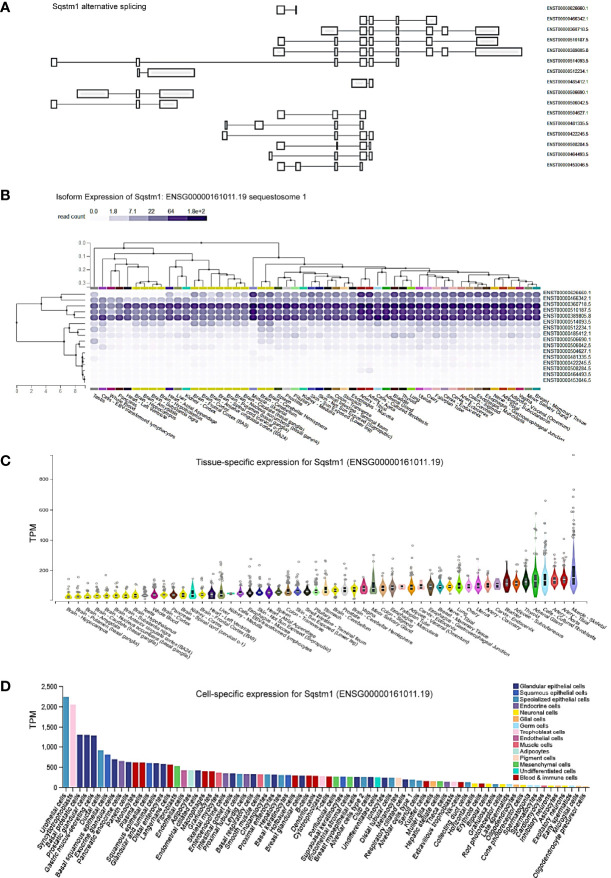
Tissue- and cell-specific expression of Sqstm1. **(A)** Sixteen splicing variants of Sqstm1 primary transcript, nine of which encode proteins, with the dominant transcript ENST00000389805 encoding a protein of 440 aa in length. **(B, C)** Tissue- and cell-specific expression of *Sqstm1* splicing variants, respectively. Total transcript of *Sqstm1* gene (ENSG000000161011) is shown. **(D)** Cell-specific expression of Sqstm1 transcript. **(A–C)** are the results from GTEx portal, and **(D)** from ProteinAtlas portal. TPM, transcripts per million; NX, denoted normalized expression. Read counts and TPM values were generated with RNA-SeQC v1.1.9 (38).

To understand the role of p62 (also known as SQSTM1) in different contexts, we first evaluated its tissue- and cell-specific expression patterns in humans, in GTEx and ProteinAtlas portals, which include datasets obtained from human protein atlas (HPA), functional annotation of the mammalian genome (FANTOM v5), and GTEx projects. The p62-encoding gene *Sqstm1* produces sixteen alternatively spliced variants (**Figure 1A**). The ENSEMBL portal shows that nine of these sixteen splice variants encode proteins, with the size of 440 aa for the dominant transcript ENST00000389805. Analysis in GTEx portal indicates that five of the splice variants are widely expressed in various tissues and cell lines (**Figure 1B**), with the highest levels being culminated in skeletal muscle, adrenal gland, and cultured fibroblasts (**Figures 1B, C**).

Regarding cell-specific expression, analysis in ProteinAtlas portal shows that the transcript abundance of p62 is mainly enriched in epithelial cells from different tissues (**Figure 1D**).

### p62 Is Upregulated in LIHC

**Figure 2:**
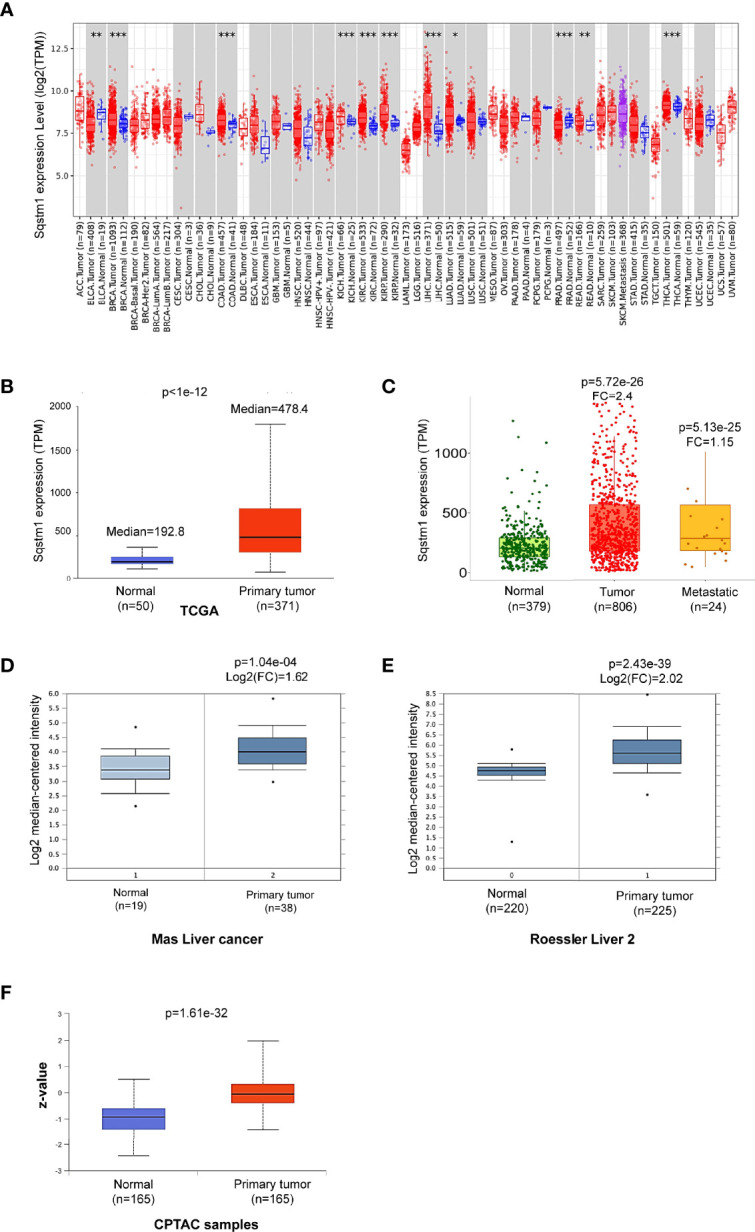
Sqstm1 is upregulated in LIHC. **(A)** Deregulation of Sqstm1 at the transcriptional level in various cancers (*p < 0.05; **p < 0.01; ***p < 0.001). Methodology was described in detail in (20, 21). Blue: Normal tissues; Red: Tumor tissues. Pink: Tumor tissues without normal tissue controls; Purple: Metastasis tumor tissues. ACC, Adrenocortical carcinoma; BLCA, Bladder Urothelial Carcinoma; BRCA, Breast invasive carcinoma; CESC, Cervical squamous cell carcinoma and endocervical adenocarcinoma; CHOL, Cholangio carcinoma; COAD, Colon adenocarcinoma; DLBC, Diffuse large B-cell lymphoma; ESCA, Esophageal carcinoma; GBM, Glioblastoma multiforme; HNSC, Head and Neck squamous cell carcinoma; KICH, Kidney chromophobe; KIRC, Kidney renal clear cell carcinoma; KIRP, Kidney renal papillary cell carcinoma; LAML, Acute myeloid leukemia; LGG, Brain lower-grade glioma; LIHC, Liver hepatocellular carcinoma; LUAD, Lung adenocarcinoma; LUSC, Lung squamous cell carcinoma; MESO, Mesothelioma; OV, Ovarian serous cystadenocarcinoma; PAAD, Pancreatic adenocarcinoma; PCPG, Pheochromocytoma and paraganglioma; PRAD, Prostate adenocarcinoma; READ, Rectum adenocarcinoma; SARC, Sarcoma; SKCM, Skin cutaneous melanoma; STAD, Stomach adenocarcinoma; TGCT, Testicular germ cell tumors; THCA, Thyroid carcinoma; THYM, Thymoma; UCEC, Uterine corpus endometrial carcinoma; UCS, Uterine carcinosarcoma; UVM, Uveal melanoma. **(B–E)** Sqstm1 transcription is upregulated in LIHC. **(F)** Sqstm1 protein level is upregulated in LIHC. **(A)** is the results from TIMER2 portal, **(B, C)** from UALCAN, **(D, E)** from Oncomine, and **(F)** from UALCAN. Median Value = median(logData); Median-centered intensity= logData-Median Value. Z-values represent standard deviations from the median across LIHC samples.

We next analyzed p62 mRNA abundance in different human cancers, using Oncomine, TIMER2.0, GEPIA2, UALCAN, and DriverDBv3. Results from different portals show that *Sqstm1* transcript is significantly upregulated in LIHC, breast cancer (BRCA), colon adenocarcinoma (COAD), kidney cancers, and thyroid carcinoma (THCA), and downregulated in bladder urothelial carcinoma (BLCA) and prostate adenocarcinoma (PRAD), compared with the levels in corresponding normal (healthy) tissues. Results from the TCGA dataset in TIMER2 are shown in **Figure 2A**.

Analysis of the TCGA dataset in UALCAN shows that p62 mRNA level is 2.8-fold higher (p<1e-12) in primary LIHC (n=371) compared to normal liver tissues (n=50) (**Figure 2B**). Analysis of gene chip data from a combination of different datasets in TNMPlot, including the TCGA, GEO, GTEx, and TARGET datasets, indicates that p62 mRNA level is 2.40-fold higher in primary LIHC (n=806; p=5.72e-26), and is marginally higher (1.15-fold) in metastatic LIHC (n=24; p=5.13e-25), compared to the normal (n=379) (**Figure 2C**). Significant upregulation of p62 is consistently detected at other algorithm platforms with various datasets, including Mas liver cancer dataset and Roessler liver cancer 2 dataset in Oncomine (**Figures 2D, E**). Correspondingly, analysis of CPTAC samples indicates that p62 protein level is also significantly upregulated in LIHC (**Figure 2F**). Together, these analyses of various datasets reveal that SQSTM1/p62 is significantly upregulated at both transcriptional and translational levels in liver cancer patients.

**Figure 3:**
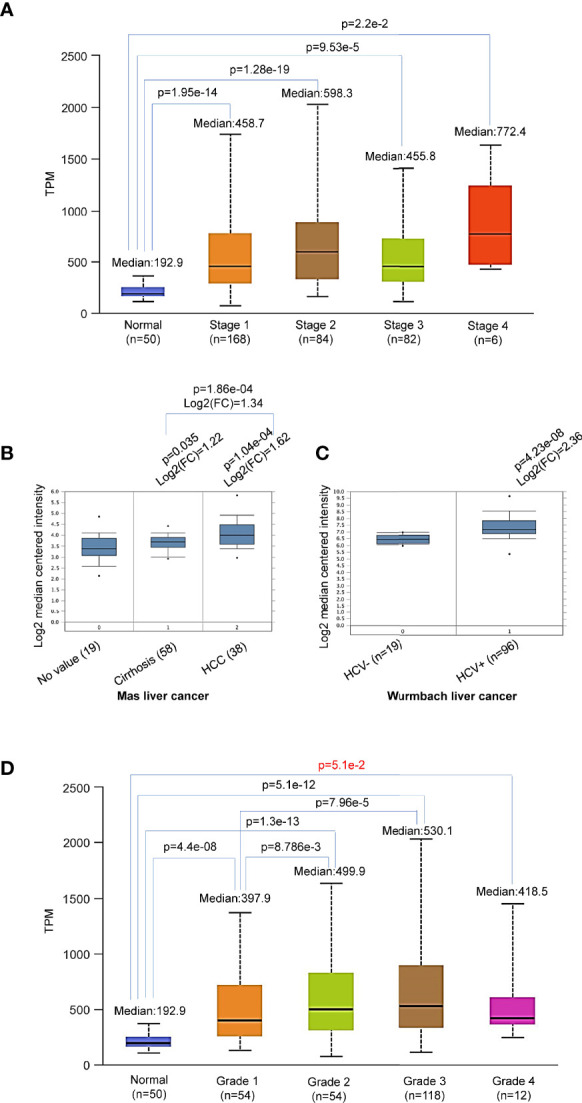
Differential expression of Sqstm1 in liver cancer stages and grades. **(A, B)** Sqstm1 is upregulated as early as liver cirrhosis and through all stages. **(C)** HCV infection is significantly associated with Sqstm1 upregulation. **(D)** Differential upregulation of Sqstm1 in clinical stages of LIHC. p value in red is not significant. **(A)** is the results from UALCAN portal, **(B, C)** from Oncomine, and **(D)** from UALCAN. Median Value = median(logData); Median-centered intensity= logData-Median Value.

Further analysis shows that p62 upregulation occurs at all stages of LIHC (**Figure 3A**), starting from as early as liver cirrhosis (**Figure 3B**). However, there are no significant differences between each two stages (**Figure 3A**), except a significant difference (p=1.86e-04) between liver cirrhosis (n=58) and liver cancer (n=38) (**Figure 3B**). Analysis of Wurmbach liver cancer dataset shows that HCV infection plays a significant role in p62 upregulation, with log2(FC)=2.36 and p=4.23e-08 comparing HCV-positive patients (n=96) to HCV-negative patients (n=19) (**Figure 3C**). Furthermore, p62 levels are significant different between tumor grades 1 and 2, and between grades 1 and 3 (**Figure 3D**). The difference between grade 4 and any other grade or normal is not significant, likely due to the small sample size (n=12) (**Figure 3D**). In addition, p62 levels are significant different in different races of liver cancer patients, with Caucasian patients (n=177) have the lowest p62 levels, compared to Africans and Asians (n=17, p= 3.91e-02, and n=157, p=2.74e-02, respectively).

In summary, p62 is accumulated during the cancer progress in LIHC, starting with a significant upregulation from liver cirrhosis, and HCV infection makes a significant contribution.

### p62 Gene Undergoes a Low Rate of Mutations in LIHC

**Figure 4:**
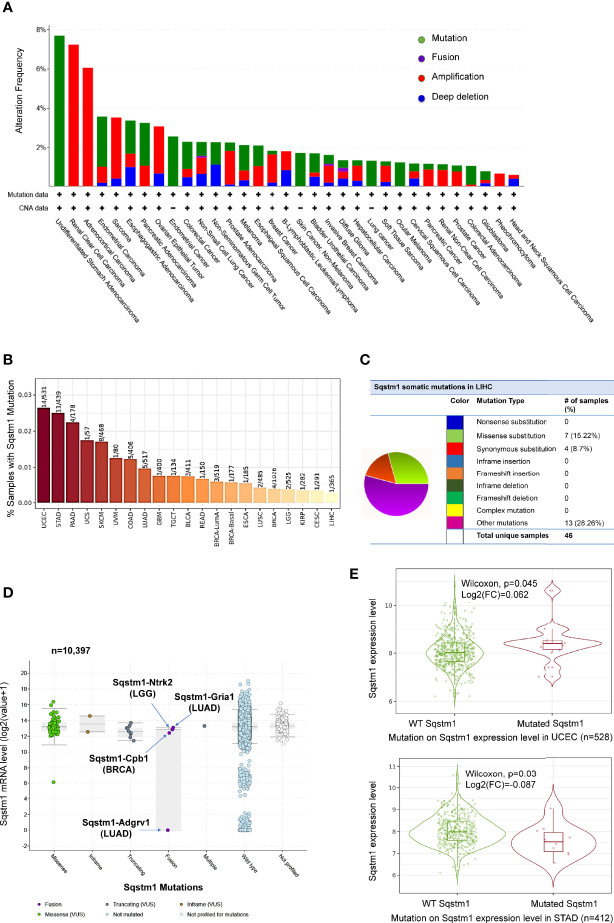
Profile of *Sqstm1* gene mutation in cancers. **(A, B)** Frequencies of *Sqstm1* gene mutation in various cancers. **(C)** Frequencies of *Sqstm1* somatic mutations in LIHC. **(D)** *Sqstm1* gene fusion and its effect on Sqstm1 expression in various cancers. **(E)** Representative results from UCEC and STAT showing the association of *Sqstm1* mutation with its expression level. **(A)** is the results from cBioportal, **(B)** from TIMER2, **(C)** from COSMIC, **(D)** from cBioportal, and **(E)** from TIMER2. FC, Fold change (the differential expression levels between samples with WT and mutated *Sqstm1*).

LIHC is very heterogeneous, with over 28,000 different somatic mutations of a large range of genes having been identified. Mutation analysis of TCGA dataset in TIMER, cBioportal, and DriverDBv3 portals shows that the *Sqstm1* gene undergoes near 8% of point mutation, followed by amplification and deep deletion, in various cancers (**Figure 4A**). However, contrast to the genes encoding Keap1 and NRF2 that among top genes undergo mutations in LIHC, *Sqstm1* only displays a low rate of mutations in LIHC (1/365), whereas undergoes higher mutation rates in UCEC (14/531), STAD (11/439), and PAAD (4/178) (**Figure 4B**). However, COSMIC portal shows that *Sqstm1* cDNA somatic mutations (substitutions and others) were detected in 24 out of 46 LIHC samples (52.17%) (**Figure 4C**).

FusionGDB analysis reveals 33 different *Sqstm1* fusion genes in various cancers and diseases (**Supplementary Table S1**). cBioportal analysis shows that *Sqstm1-Adgrv1* (not detected in FusionGDB) and *Sqstm1-Gria1* fusions occur in LUAD, *Sqstm1-Cpb1* fusion occurs in BRCA, and *Sqstm1-Ntrk2* occurs in LGG, with *Sqstm1-Adgrv1* fusion in LUAD associated with a remarkable decrease of Sqstm1 expression (**Figure 4D**). *Usp10*-*Sqstm1* fusion was found in a patient with combined hepatocellular and intrahepatic cholangiocarcinoma. Consistent results from different portals, including cBioportal and TIMER2, indicate that the overall *Sqstm1* mutation has significant effects on its expression in UCEC (increase, p=0.045) and STAD (decrease, p=0.03) (**Figure 4E**). Analysis was not performed for other types of cancer (including LIHC) in that the sample sizes with *Sqstm1* mutation of these cancers are not powerful enough (**Figure 4B**).

Together, these findings support the claim that the deregulation of p62 expression in different cancers results from both epigenetic reprogramming and gene mutation. As one of these epigenetic mechanisms, *Sqstm1* gene transcription is transactivated by diverse transcription factors, including NRF2 that is activated in response to oxidative stress, NFκB, Ets/Pu.1, Myc, among many others (12). Importantly, we have collected solid evidence showing that p62 expression is induced by Epstein-Barr Virus (EBV) principal oncogenic product Latent Membrane Protein 1 (LMP1) in EBV latency, supporting a specific role for p62 in EBV-mediated cancers (39).

### Mutation of Top Genes in Correlation With p62 Upregulation in LIHC

**Figure 5:**
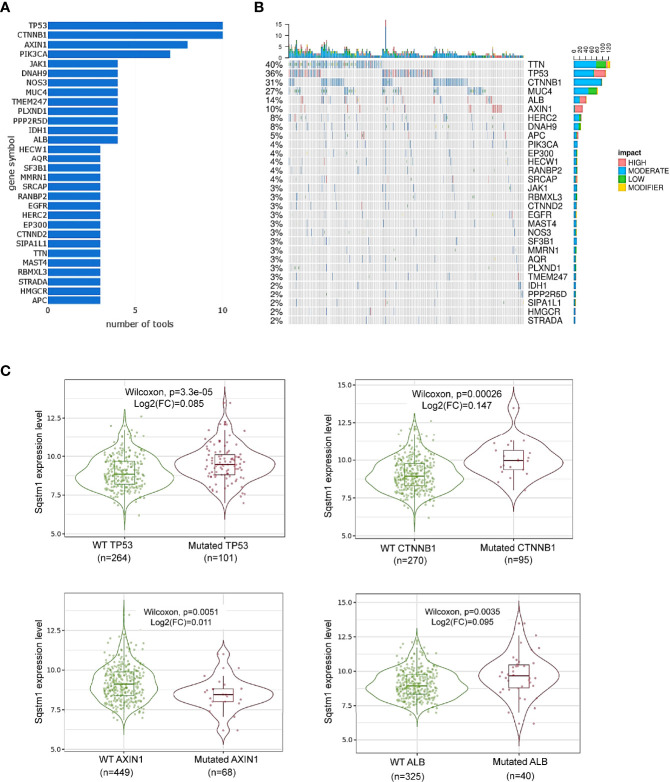
Association of Sqstm1 expression level with gene mutation. **(A, B)** Top 30 genes that are mutated in LIHC. “number of tools” in **(A)** means the number of bioinformatic algorithms/tools were used to integrate multiomics to address the cancer driver events at distinct molecular levels (30). The percentage in **(B)** represents the total percentage of mutation in the samples for each mutated gene. The x-axis in **(B)** represents the samples from different LIHC patients. **(C)** Representative genes whose mutation is significantly associated with Sqstm1 expression in LIHC. **(A, B)** are the results from cBioportal, and **(C)** from TIMER2. FC, Fold change (the differential expression levels between samples with WT and mutated *Sqstm1*).

DriveDBv3 analysis shows that, in agreement with previous reports (40, 41), top genes mutated in liver cancer include TP53, CTNNB1, AXIN1, PIK3CA, JAK1, among many others (**Figure 5A**). The effects of mutation of top 30 genes on their own expression are shown in **Figure 5B**.

Among these mutated genes, TIMER2 analysis revealed that mutation of TP53, CTNNB1 (encoding β-catenin), or ALB (encoding serum albumin) significantly correlates with, and mutation of AXIN1 (a negative regulator of β-catenin stability) reversely correlates with, the p62 expression level (p<0.05) (**Figure 5C**). However, mutations of the genes encoding NRF2 (NFE2L2), Keap1, TTN, MUC4, PIK3CA, or JAK1, are not significantly correlated with p62 level (**Supplementary Figure 1**). Mutation of other genes on this list may be associated with p62 deregulation, but the sample sizes of their mutation are not powerful for statistical analysis.

In addition to these frequently mutated genes, we further analyzed whether mutations of any other genes are associated with p62 deregulation in LIHC in muTarget portal. 25 genes (including most of the above frequently mutated genes), such as DOCK2, TG, and DNAH10, whose mutations were found to be significantly correlated with p62 deregulation (p<0.05; fold change>1.44) (**Supplementary Table S2**).

### p62 Promoter Methylation Is Downregulated in LIHC

**Figure 6:**
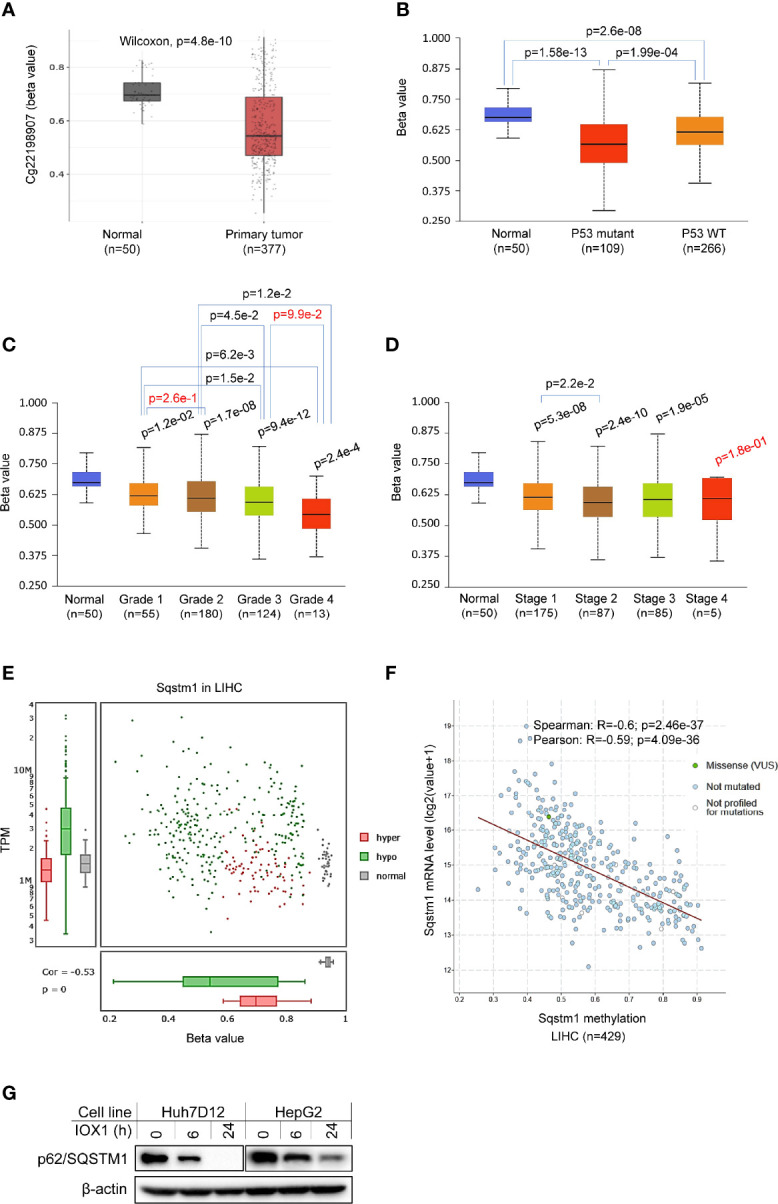
Association of Sqstm1 expression level with its promoter methylation. **(A)** The *Sqstm1* gene promoter is significantly demethylated in LIHC. **(B)** p53 is association with *Sqstm1* gene promoter demethylation. **(C)** The *Sqstm1* gene promoter is significantly demethylated in all stages. **(D)** The *Sqstm1* gene promoter is gradually demethylated through the cancer progress. **(E)** Analysis of hyper- and hypo-methylation of the *Sqstm1* gene promoter. **(F)** The *Sqstm1* promoter methylation is reversely correlated with its expression. p values in red are not significant. **(A)** is the results from SAMRT portal, **(B–D)** from UALCAN, E from DriverDBv3, and F from cBioportal. **(G)** Experimental validation of demethylation in regulation of SQSTM1 expression. Huh7D12 and HepG2 cells were treated with 0.3 mM of the demethylase inhibitor IOX1 for indicated hours, followed by immunoblotting.

Promoter methylation analysis of TCGA dataset in SMART portal indicates that the *Sqstm1* gene promoter is significantly demethylated (the chromosome 5 region spanning nucleotides 179805165~179837098) in LIHC (p=3.76e-12. **Figure 6A**), and more demethylation of the *Sqstm1* gene promoter was found in patients with p53 mutation (p=1.99e-4. **Figure 6B**). Interestingly, the downregulation of *Sqstm1* gene promoter methylation is progressed with the tumor grades (**Figure 6C**), and also with the stages (**Figure 6D**). Further analysis in DriverDBv3 shows that only 0.777% hypo methylation (beta value 0.25~0.3) and 0.223% hyper methylation (beta value 0.5~0.7) of the *Sqstm1* gene promoter methylation exist in LIHC and the methylation reversely correlates with its expression (Spearman correlation coefficient: -0.53, p=0) (**Figure 6E**). Analyses in cBioportal further confirm that the *Sqstm1* promoter methylation is reversely correlated with *Sqstm1* expression (**Figure 6F**). We further experimentally validated that treatment of liver cancer cell lines with the demethylase inhibitor IOX1 downregulates the SQSTM1/p62 protein level (**Figure 6G**). These findings indicate that *Sqstm1* gene promoter demethylation significantly correlates with the upregulation of p62 expression in LIHC, suggesting that promoter demethylation plays an important role in upregulating p62 expression in LIHC.

### p62 Genome-Wide Association Patterns in LIHC

**Figure 7:**
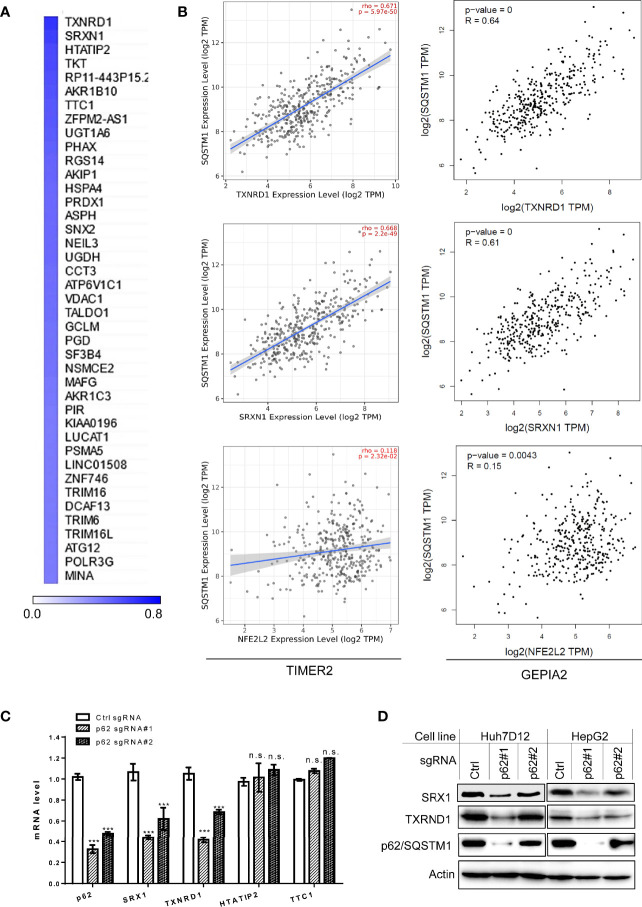
Sqstm1-associated molecular signature in LIHC. **(A)** Top 40 genes correlated with Sqstm1 at the expression level in LIHC, with Pearson correlation coefficient values (PCC) >0.5. **(B)** Validation of individual genes in association with Sqstm1 at the transcriptional level. Results were obtained from TIMER2 (left) and GEPIA2 (right). **(C, D)** p62/Sqstm1 depletion downregulates SRX1 and TXNRD1 expression at both transcriptional and translational levels. In qPCR results, the mRNA level of each sample with control sgRNA was set to 1. ***p < 0.001. n.s., not significant.

Next, we performed genome-wide association studies (GWAS) to profile p62-associated molecular signatures in LIHC, in GEPIA2, Oncomine, TNMPlot, and UALCAN portals. Results have identified a pool of genes that correlate with p62 (PCC>0.5) at the mRNA level in LIHC, such as TXNRD1, SRXN1, NFE2L2, TTC1 (encoding TRP1), and TKT (**Supplementary Table S3**). Representative results from GEPIA2 portal are shown in **Figure 7A**. We validated the correlation of p62 with selected genes in both TCGA and GTEx lung cancer datasets, by one-to-one paired analysis in GEPIA2 and/or TIMER2 portals.

Representative results for the key regulators of oxidative stress and metabolism are shown in **Figure 7B**, including TXNRD1 (encoding the Thioredoxin reductase TrxR1), the reductase-encoding genes SRXN1 (encoding SRX1), HTATIP2 (encoding HIV1 TAT-Interactive Protein 2), AKR1b10 (encoding a member of the aldo/keto reductase superfamily), and TKT (encoding transketolase), as well as NFE2L2 (encoding the master antioxidant transcription factor NRF2) that governs the antioxidant stress in various cancers through the Keap1-NRF2-p62 pathway. SRX1 is known to contribute to oxidant stress resistance in various cancers by controlling the activity of a subgroup of PRDXs (42, 43). Consistent with our findings, TXNRD1 was found to play a decisive role in hepatocellular carcinoma malignancy (44).

We further employed “loss-of-function” assays to assess which of four selected p62-correlated genes, including TXNRD1, SRXN1, HTATIP2, and TTC1, are deregulated by p62 in LIHC. To this end, we depleted p62 in in two liver epithelial cancer cell lines, Huh7D12 and HepG2, using CRISPR/Cas9-mediated approach with p62-specific sgRNA plasmids that were proven to be successful to downregulate p62 expression in our recent publication (39). qPCR and immunoblotting analyses revealed that SRX1 and TXNRD1, but not HTATIP2 or TTC1, are targeted by p62-mediated mechanisms at the transcriptional and translational levels (**Figures 7C, D**).

### High p62 Levels Correlate With Severe Prognosis of LIHC Patients

**Figure 8:**
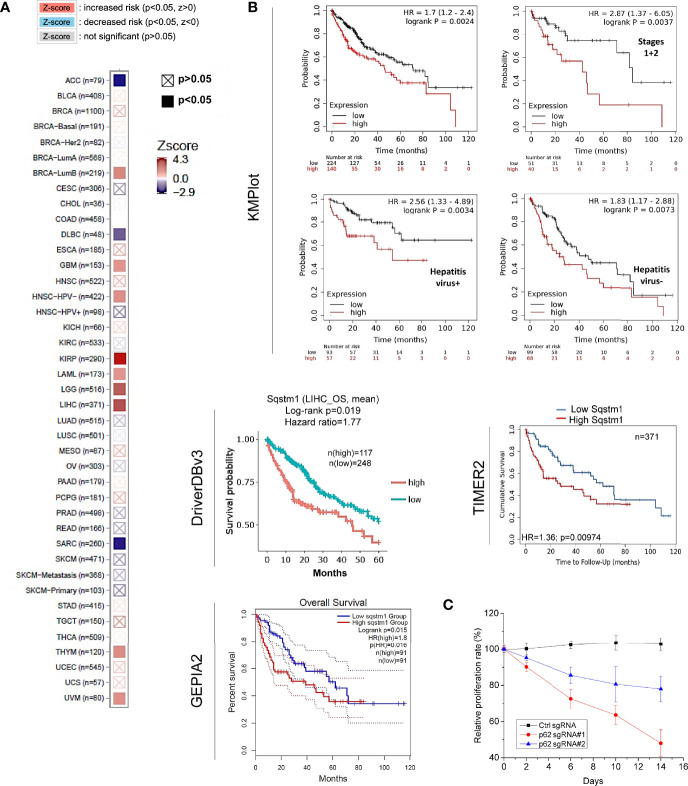
Sqstm1 expression is associated with the overall survival of certain cancers. **(A)** Sqstm1 expression level is associated with the overall survival (OS) of certain cancer patients. **(B)** Sqstm1 expression level reversely correlates with the OS of LIHC patients. **(A)** is results from TIMER2, and **(B)** from different portals showing the consistency. For KMPlot, the settings were: JetSet best probe set, excluding outlier arrays, and univariate. **(C)** p62/Sqstm1 depletion reduces the proliferation of the liver cancer cell line Huh7D12. The proliferation rate of the cells with control sgRNA at each time point was set to 100%.

Regarding the clinical outcomes of p62 deregulation in cancers, we first assessed the prognosis value of p62 deregulation across various cancers. The relation between p62 transcription levels and the overall survival (OS) rates of TCGA cancer patients was analyzed in TIMER2. Results show that the abundance of p62 reversely correlates with the OS of KIRP (z=4.31, p=0.00016), LIHC (z=4.03, p=0.00056), LGG (z=3.77, p=0.00016), amongst several other cancers (**Supplementary Table S4**), and positively correlates with the OS of ACC (z=-2.862, p=0.00421), SARC (z=-2.84, p=0.004473), and DLBC (z=-2.11, p=0.03448) (**Figure 8A**).

The reverse correlation of p62 upregulation with the OS of LIHC patients was further confirmed in TIMER2, GEPIA2, and DriverDBv3 portals with TCGA datasets, and in Kaplan Meier plotter (KMPlot) portal with TCGA, EGA, and GEO datasets (**Figure 8B**). Consistent with the results that HCV infection contributes to p62 upregulation (**Figure 3C**), the p62 level and the OS have a greater correlation in hepatitis virus-positive LIHC patients (p=0.0034) compared to hepatitis virus-negative patients (p=0.0073) (**Figure 8B**).

More importantly, we show that depletion of p62 in HepG2 liver cell line dramatically reduces cell proliferation (**Figure 8C**). Similar results were also obtained in the liver cell line Huh7D12 (not shown).

Together, these results indicate that p62 can serve as a promising prognostic biomarker for LIHC.

### Possible Mechanism Underneath Tumor-Suppressing Function of p62 in LIHC

Accumulating evidence shows that p62 plays multifaceted roles in LIHC, including its ability to promote LIHC initiation by activating NRF2, mTORC1, and c-MYC pro-oncogenic pathways in hepatocytes (2).

**Figure 9:**
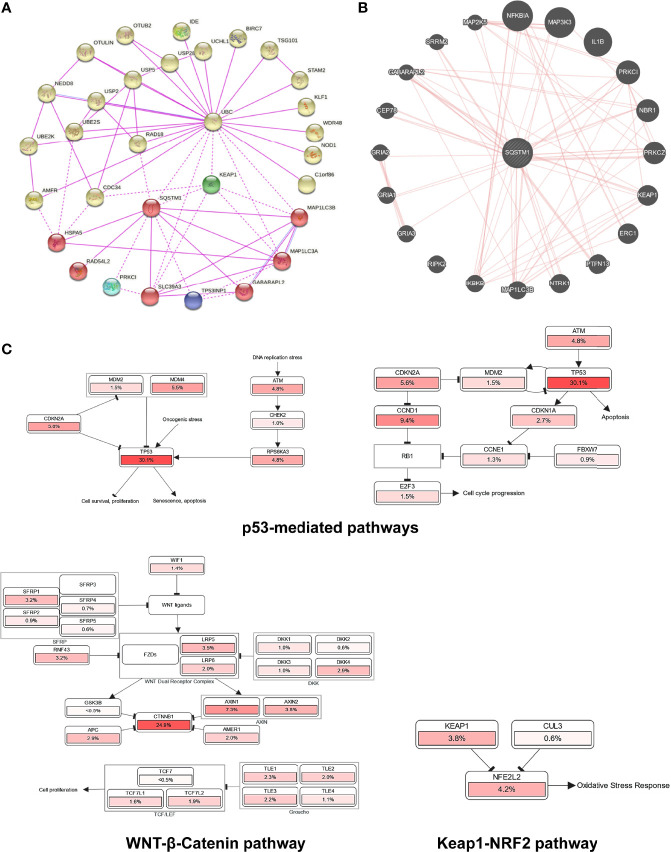
Genome-wide interaction and pathway association of Sqstm1 in LIHC. **(A)** Known SQSTM1 protein physical interaction network, as analyzed in STRING databases. Co-expression, textmining, neighborhood, and gene fusion were excluded in the settings. **(B)** Physical interactors of SQSTM1 protein as identified in BioGRID with various databases from both high and low throughput assays. **(C)** The pathways that are deregulated in correlation with SQSTM1 in LIHC. The deregulation of individual genes is shown in red. The more frequently a given gene is deregulated, the deeper red is shown.

To explore potential novel mechanisms responsible for p62-mediated tumor promoting function in LIHC, we analyzed genome-wide p62 interaction network in BioGRID, STRING, and GeneMANIA. Results show that a large pool of p62 interactors in both high and low throughput profiling assays (**Supplementary Table S5**), including many involved in the autophagy and ubiquitin systems such as UBC, LC3 (MAP1LC3A/B), ULK1, USPs, UBEs, AMFR (RNF45), and OTULIN and OTUB2; Keap1 and NRF2 that are components of the master antioxidative pathway; and GRIAs (Ionotropic glutamate receptors) that are involved in excitatory synaptic transmission in the central nervous system (**Figures 9A, B**).

Finally, we analyzed the possible pathways mediated by p62 in Pathcard, KEGG and Uniport, and those associated with p62 upregulation in LIHC in cBioportal. In agreement with its tumor-promoting role in LIHC, the p62 level in LIHC has a remarkable association with p53-mediated cell cycle regulation and cell survival/proliferation, WNT/β-Catenin-mediated cell proliferation, and Keap1-NRF2 antioxidative stress (**Figure 9C** and **Supplementary Figure 2**).

## Discussion

We show in this study that: 1) p62 is significantly upregulated in several malignancies, including LIHC (**Figure 2**); 2) the upregulation of p62 transcription starts as early as liver cirrhosis, and HCV infection makes a significant contribution to p62 upregulation (**Figure 3**); 3) the demethylation of p62-encoding gene (*Sqstm1*) promoter, but not its mutations that occur at low rates in LIHC, makes a significant contribution to its upregulation in LIHC (**Figure 6**); 4) genome-wide association analyses identified p62-associated molecular signature in LIHC, and SRX1 and TXNRD1 are further confirmed to be targeted by p62 at transcriptional and translational levels (**Figure 7**). 5) Higher p62 levels are significantly associated with worse prognosis of LIHC (**Figure 8**). 6) Mechanistically, p62 expression level reversely correlates with the deregulation of p53-mediated cell survival and cell cycle progress, and correlates with Keap1-NRF2-mediated antioxidative stress and Wnt/β-Catenin-mediated cell proliferation (**Figure 9**). Surprisingly, our analysis indicates that p62 expression level is poorly associated with the frequencies of tumor infiltrating lymphocytes (cutoff: R>0.3; p<0.05. **Supplementary Figure 3**), although it plays a crucial role in the tumor microenvironment in different cancer contexts (8, 45), and induces cancer-associated fibroblast activation in LIHC (46).

It is well known that the transcription factor NRF2, among many others (12), transactivates p62 gene expression in response to oxidative stress in various contexts (47). Importantly, our study implicates novel mechanisms for p62 upregulation in LIHC, including the demethylation of the *Sqstm1* gene promoter, and the transcription factors p53 (loss-of-function mutation in LIHC) and β-Catenin/TCF. By analyzing the *Sqstm1* gene promoter, we identified multiple potential p53-binding sites (**Supplementary Figure 4**). In agreement with our findings, p62 was reported to be transcriptionally suppressed by the β-Catenin/TCF pathway (48), and in turn, autophagy negatively regulates the Wnt/β-Catenin/TCF pathway at least by targeting β-Catenin for degradation (49), in cancer cells. We will validate the transcriptional regulation of p62 by p53 and the Wnt/β-Catenin/TCF pathway in LIHC in separate projects.

p62 is a multifunctional protein that plays important roles in both cytoplasmic and nuclear compartments. In the cytoplasm, it is well known to act as a selective autophagy receptor, a ubiquitin sensor, and a signal transducing hub, which is involved in NRF2-mediated antioxidative defense, mTORC1-mediated nutrient sensing and metabolic reprogramming, cGAS-STING-mediated antitumor immunity, NFκB activation, inflammation, and apoptosis. In the nucleus, p62 regulates DNA damage response, proteasomal activity, and the assembly of PML bodies (2, 12, 50). Of note, our study has identified SRX1 and TXNRD1 as targets of p62 that are regulated by p62 at both mRNA and protein levels. The mechanism may involve p62-mediated activation of the transcription factors NFκB, NRF2, and STATs. In support of this possibility, SRX1 is a known NRF2 transcriptional target (51). TXNRD1 cooperates with Keap1 in sensing cellular stresses to modulate NRF2 activity, and plays a pivotal role in redox (reduction-oxidation) homeostasis related to the glutathione (GSH) and thioredoxin (Trx) systems that are mediated by NADPH-dependent disulfide reductases (52).

The protein p62 can be extensively modified at the post-translational level by site-specific phosphorylation, ubiquitination, acetylation, and others in different functional contexts (2, 12). For example, for human p62, S403 phosphorylation and K420 ubiquitination of p62 are involved in its autophagy function, S349 phosphorylation is required for its regulation of the Keap1-NRF2 pathway activity in a feedback loop, and T269/S272 phosphorylation is required for its cyto-nuclear shuttling. p62 itself is targeted by selective autophagy for degradation that requires its ubiquitination.

Our study supports the claim that p62 is a crucial cancer promoter that is significantly upregulated in LIHC, and could serve as a diagnostic and prognostic marker. Although p62 has previously implicated in LIHC, our comprehensive big data analyses disclose potential novel mechanisms underlying p62 regulation and its potential roles in this cancer context. Further experimental validation of these mechanisms is of importance for better understanding the interaction of p62 with LIHC.

**Supplementary Tables S1** **-** **S5** and **Figures S1** **-** **S4** are available online.

## Data Availability Statement

The original contributions presented in the study are included in the article/**Supplementary Material**. Further inquiries can be directed to the corresponding author.

## Author Contributions

Conceptualization: LW and SN; Data analysis: SN, CH, and LW; Funding acquisition: LW and SN; Methodology: LW, MH, CH, and SN. Writing and editing: LW and SN. All authors have read and agreed to the published version of the manuscript.

## Funding

This work was supported by NIH, CA252986 (LW), DE029621 (SN) and ASH (SN), and in part by the NIH grant C06RR0306551.

## Conflict of Interest

The authors declare that the research was conducted in the absence of any commercial or financial relationships that could be construed as a potential conflict of interest.

## Publisher’s Note

All claims expressed in this article are solely those of the authors and do not necessarily represent those of their affiliated organizations, or those of the publisher, the editors and the reviewers. Any product that may be evaluated in this article, or claim that may be made by its manufacturer, is not guaranteed or endorsed by the publisher.
