# Supplementary figures and images for: Bioinformatics-Driven Identification of p62 as A Crucial Oncogene in Liver Cancer
Source: Front Oncol. 2022 Jun 24;12:923009. doi: 10.3389/fonc.2022.923009 (PMC9263135; doi:10.3389/fonc.2022.923009)

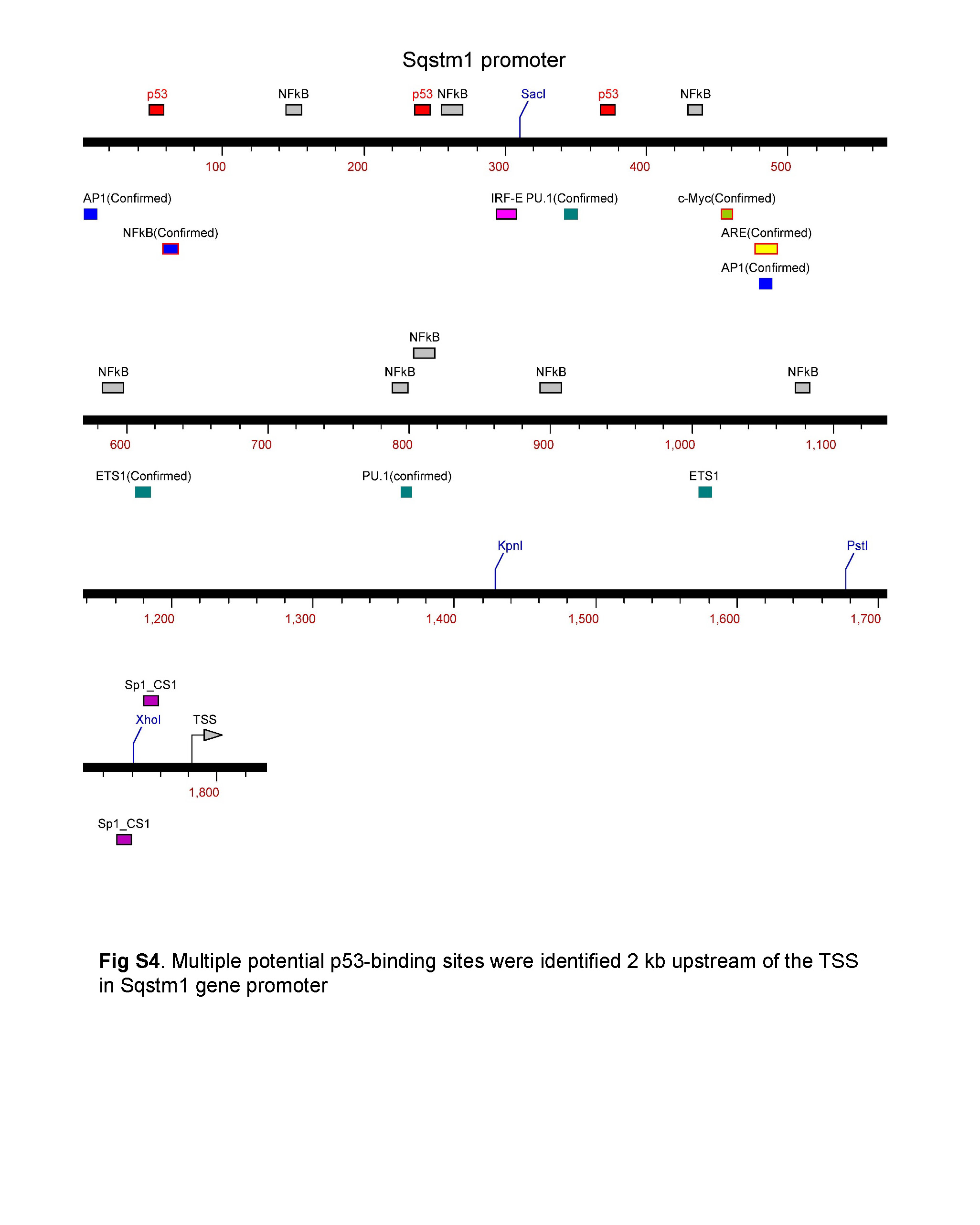

Supplement: Supplementary file 4 [file Image_4.jpg]
